# Molecular Basis of Mismatch Repair Protein Deficiency in Tumors from Lynch Suspected Cases with Negative Germline Test Results

**DOI:** 10.3390/cancers12071853

**Published:** 2020-07-09

**Authors:** Alisa Olkinuora, Annette Gylling, Henrikki Almusa, Samuli Eldfors, Anna Lepistö, Jukka-Pekka Mecklin, Taina Tuulikki Nieminen, Päivi Peltomäki

**Affiliations:** 1 Department of Medical and Clinical Genetics, University of Helsinki, 00014 Helsinki, Finland; annette.h.gylling@gmail.com (A.G.); taina.nieminen@helsinki.fi (T.T.N.); paivi.peltomaki@helsinki.fi (P.P.); 2 Institute for Molecular Medicine Finland (FIMM), University of Helsinki, 00014 Helsinki, Finland; henrikki.almusa@helsinki.fi (H.A.); seldfors@mgh.harvard.edu (S.E.); 3 Department of Gastrointestinal Surgery, Helsinki University Hospital and University of Helsinki, 00290 Helsinki, Finland; anna.lepisto@hus.fi; 4 Department of Surgery, Jyväskylä Central Hospital, 40620 Jyväskylä, Finland; jukka-pekka.mecklin@ksshp.fi; 5 Faculty of Sports and Health Sciences, University of Jyväskylä, 40014 Jyväskylä, Finland

**Keywords:** Lynch syndrome, DNA mismatch repair, colorectal cancer, deep sequencing

## Abstract

Some 10–50% of Lynch-suspected cases with abnormal immunohistochemical (IHC) staining remain without any identifiable germline mutation of DNA mismatch repair (MMR) genes. MMR proteins form heterodimeric complexes, giving rise to distinct IHC patterns when mutant. Potential reasons for not finding a germline mutation include involvement of an MMR gene not predicted by the IHC pattern, epigenetic mechanism of predisposition, primary mutation in another DNA repair or replication-associated gene, and double somatic MMR gene mutations. We addressed these possibilities by germline and tumor studies in 60 Lynch-suspected cases ascertained through diagnostics (*n* = 55) or research (*n* = 5). All cases had abnormal MMR protein staining in tumors but no point mutation or large rearrangement of the suspected MMR genes in the germline. In diagnostic practice, MSH2/MSH6 (MutS Homolog 2/MutS Homolog 6) deficiency prompts *MSH2* mutation screening; in our study, 3/11 index individuals (27%) with this IHC pattern revealed pathogenic germline mutations in *MSH6*. Individuals with isolated absence of MSH6 are routinely screened for *MSH6* mutations alone; we found a predisposing mutation in *MSH2* in 1/7 such cases (14%). Somatic deletion of the *MSH2*-*MSH6* region, joint loss of MSH6 and MSH3 (MutS Homolog 3) proteins, and hindered MSH2/MSH6 dimerization offered explanations to misleading IHC patterns. Constitutional epimutation hypothesis was pursued in the MSH2 and/or MSH6-deficient cases plus 38 cases with MLH1 (MutL Homolog 1)-deficient tumors; a primary *MLH1* epimutation was identified in one case with an MLH1-deficient tumor. We conclude that both *MSH2* and *MSH6* should be screened in MSH2/6- and MSH6-deficient cases. In MLH1-deficient cases, constitutional epimutations of *MLH1* warrant consideration.

## 1. Introduction

Colorectal cancer (CRC) is the third most common cancer type globally and one of the leading causes of death [1]. Lynch syndrome (LS) is the most prevalent form of hereditary predisposition to CRC and accounts for 2–3% of all CRCs [2]. LS patients also have frequent extracolonic cancers, notably endometrial cancer in females [2]. Heterozygous pathogenic variants in the DNA mismatch repair (MMR) genes, namely *MLH1, MSH2, MSH6,* and *PMS2* and less frequently, deletions in the 3′ end of *EPCAM* cause predisposition to LS [3]. Other MMR genes may contribute to cancer susceptibility with lower penetrance (*MLH3*) or predispose to polyposis (*MSH3, MLH3*) [4,5]. It is generally thought that a ‘second hit’, a somatic mutation, in the wild-type allele of the affected MMR gene is required for triggering tumorigenesis. There is, however, some evidence for haploinsufficiency for the MMR genes [6].

Based on two-hit inactivation, absence of protein expression of selected MMR gene(s) by immunohistochemical (IHC) analysis helps to direct gene testing for the purposes of LS diagnostics [7]. The human MMR mechanism functions via two interacting base proteins, MutL and MutS. Four MutL proteins (MLH1, MLH3, PMS1 and PMS2) and five MutS proteins (MSH2, MSH3, MSH4, MSH5, MSH6) have been recognized to date. Out of these, MLH1 and MSH2 are considered primary dimers, which dimerize with any of the secondary dimers in the same protein family. The absence of cellular primary dimer is typically followed by proteolytic degradation of its respective secondary dimers. Thus, IHC staining of tumor samples with *MSH2* mutations display negative nuclear staining for both MSH2 and MSH6, and tumors from individuals with *MLH1* mutations lack MLH1 and PMS2 proteins. In contrast, tumors of patients with *MSH6* mutations exhibit negative nuclei for only MSH6, and isolated absence of PMS2 proteins points to *PMS2* mutations.

The so-called universal tumor screening for LS based on IHC and/or microsatellite instability (MSI) analysis is nowadays recommended for all patients diagnosed with CRC or endometrial cancer [8,9].

IHC testing on CRC tumors has a sensitivity of 94% and specificity of 88% in the identification of patients with LS [10]. Concordance between IHC and MSI is very high in CRCs [10,11], but often lower in extracolonic tumors [6].

While IHC-based strategies generally perform well in pre-screening of LS [10,11], they are not 100% accurate. For instance, somatic MMR gene alterations, related or unrelated to the predisposing MMR gene mutations may confuse diagnostics. Somatic hypermethylation of the *MLH1* promoter [12], MMR gene mutations secondary to polymerase proofreading defects [13], and double somatic MMR gene mutations [14] are examples of mechanisms that can mislead interpretations of germline mutations. We, here, address these possible mechanisms by studies of constitutional and tumor tissues in a series of 60 LS-suspected cases with abnormal IHC but no germline mutation in the MMR genes expected to be involved according to the IHC pattern of tumors.

## 2. Results

### 2.1. Clinicopathological Characteristics and Study Design

**Figure 1:**
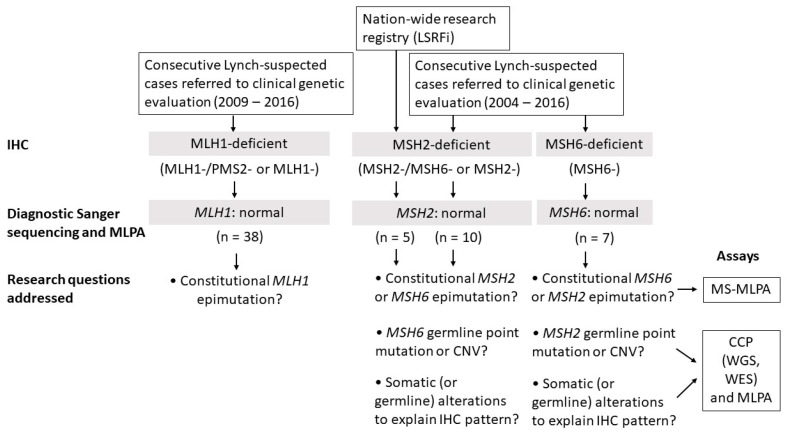
Flow chart of this investigation. Abbreviations: LSRFi, Hereditary Colorectal Cancer Registry of Finland; IHC, immunohistochemical; MS-MLPA, methylation-specific multiplex ligation-dependent probe amplification; CNV, copy number variant; CCP, Comprehensive Cancer Panel; WGS, genome sequencing; WES, exome sequencing.

Our study consisted of 60 suspected LS patients from Finland who were ascertained as described in Section 4 as well as in Figure 1. All patients were mutation negative by diagnostic testing carried out on the primary-suspect MMR gene. Clinical and molecular patient data are listed in Table S1. The average age at onset for the first LS-associated cancer (typically CRC) in all patients was 51.6 years (SD = 11.7). For MLH1, MSH2, and MSH6-deficient cohorts separately, the average ages at onset were 53.6 (SD = 13.2), 47.3 (SD = 7.7), and 50.6 (SD = 6.8), respectively.

To unravel the molecular basis of abnormal MMR protein expression in patients, three alternative scenarios were considered (Figure 1). First, the predisposing alteration may not be genetic but epigenetic. Second, the IHC pattern may not always predict the correct MMR gene. Third, absent MMR protein may be secondary to alterations in other DNA repair- or replication-related genes, or entirely somatic in origin. In the MLH1-deficient group, we concentrated on *MLH1* epimutations alone. This was because our MLH1-deficient cases originated from the time-period when exclusion of somatic *MLH1* hypermethylation was not yet part of the routine diagnostic scheme. Not knowing which cases were possibly explained by somatic *MLH1* methylation and not having tumor tissues available, further molecular dissection was not meaningful. A more comprehensive evaluation was possible for the MSH2- and MSH6-deficient cases. Laboratory assays conducted are shown in Figure 1 and case by case in Table S1. DNA methylation alterations were investigated by methylation-specific multiplex ligation-dependent probe amplification (MS-MLPA). Genetic mutation screens included panel sequencing by NimbleGen’s Comprehensive Cancer Panel (CCP) for point mutations and multiplex ligation-dependent probe amplification (MLPA) for exon-level deletions and duplications. CCP covers the entire coding regions and flanking exon/intron borders of the MMR genes plus 574 other cancer-relevant genes. In selected cases, genome-wide sequencing (such as whole-genome sequencing in the research families F70 and F88) was performed to be able to detect, for example, complex rearrangements.

All study subjects had abnormal IHC without any germline mutation in the primary MMR gene suspected based on the IHC pattern. Research questions addressed and assays used to disentangle the mechanisms behind abnormal IHC are listed at the bottom of the flow chart in Figure 1. 

### 2.2. MLH1 Cohort

**Figure 2:**
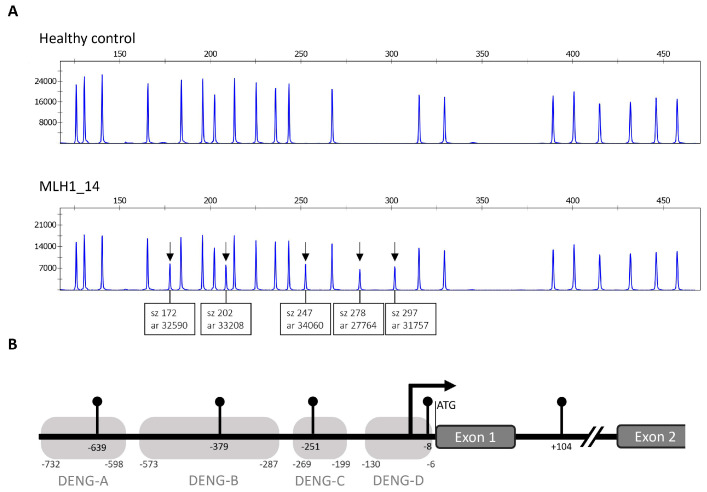
Details of constitutional epimutation affecting the *MLH1* promoter in case MLH1_14. (**A**) MS-MLPA tracing of the *MLH1* promoter region including five methylation-sensitive sites monitored in the assay. The presence of a peak at a methylation-sensitive site indicates methylation, the degree of which D_m_, the methylation dosage ratio, can be calculated from the peak areas, whereas the absence of a peak indicates no methylation (the remaining peaks shown in the figure represent reference genomic areas lacking *HhaI* sites). All five methylation-sensitive sites (arrows) are methylated in leukocyte-derived DNA from MLH1_14 and unmethylated in a healthy control. Normalized D_m_ values around 0.5 suggest that one allele is likely to be fully methylated and the other allele unmethylated in MLH1_14. (**B**) Diagram of the *MLH1* promoter region showing the positions of the methylation-sensitive sites (lollipops) relative to the translation start codon ATG (for initiating methionine). From Deng et al. [15], areas A–D are depicted, and area C closely correlates with expressional silencing. Bent arrow indicates transcription start according to Deng et al. [15].

One index case out of 38 (3%) was found positive for an apparent constitutional hypermethylation of *MLH1.* In this patient (MLH1_14 in Table S1), all five *HhaI* sites tested from the *MLH1* promoter region and flanking area showed hypermethylation (Figure 2). Methylation dosage (Dm) values ranged between 0.44 and 0.52, compatible with monoallelic methylation. The so-called Deng C region is the most important regarding transcriptional silencing by methylation [15,16]. No DNA sequence alterations were found in the immediate promoter region by Sanger sequencing, and no large rearrangements were detected by MLPA, suggesting a primary type of constitutional epimutation.

### 2.3. MSH2 Cohort

#### 2.3.1. Consecutive MSH2-Deficient Cases from Diagnostics

Genetic variants are conventionally divided into five categories of pathogenicity based on characteristics of the variant as well as the clinical and family features [3]. Variants belonging to classes 1 (benign) and 2 (likely benign) are considered harmless. Class 3 is assigned to variants of uncertain significance (VUS). Classes 4 (likely pathogenic) and 5 (pathogenic) indicate that the variant is deleterious, and clinical management protocols recommended for LS should be undertaken. Among the ten consecutive MSH2-deficient cases available for this investigation, heterozygous VUS alterations in *MSH2* were originally diagnosed for two. Case MSH2_3 had c.998G>A, p.(Cys333Tyr) variant, whereas case MSH2_10 showed c.1805T>C, p.(Leu602Pro) variant (reference sequence NM_000251.3) (Table S2). For the former variant, new data that became available while this study was ongoing, justified a shift from pathogenicity class 3 to class 5 upon assessment by the InSiGHT expert group, thus explaining MSH2-deficiency in this patient. The latter variant remains to be classified as a VUS, leaving the basis of MSH2-deficiency in MSH2_10 unsettled.

**Figure 3:**
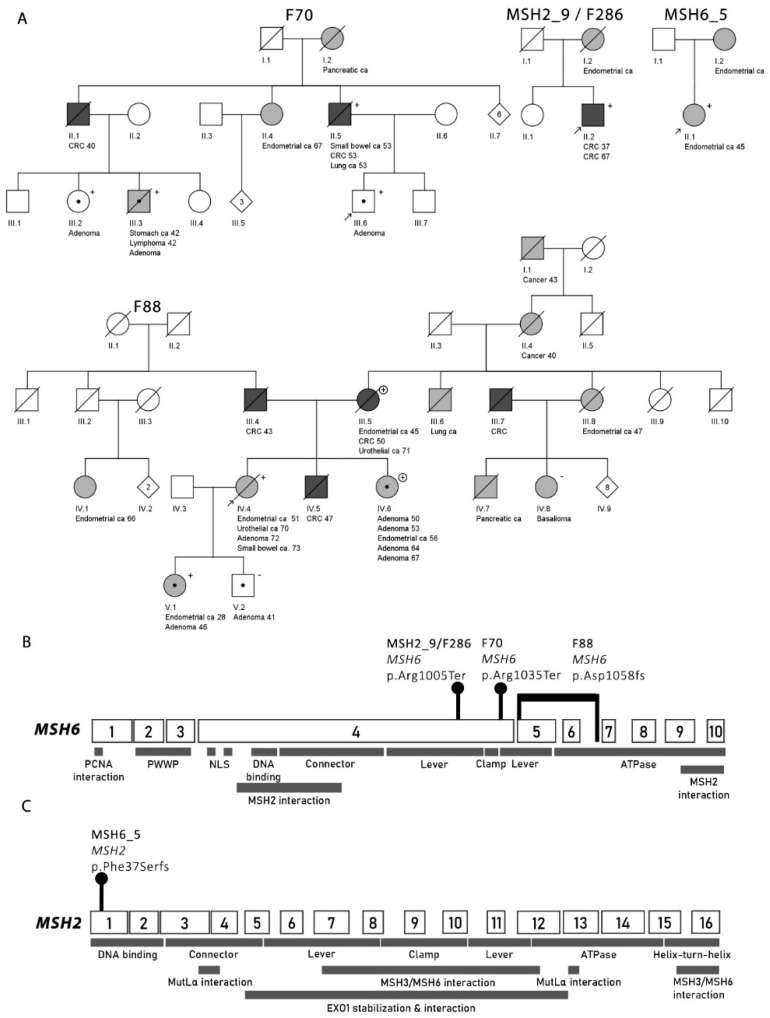
The four families that revealed a germline mutation in a mismatch repair (MMR0 gene not expected from the IHC pattern. (**A**) Pedigrees generated with Pedigree Chart Designer. Numbers below the symbols are patient identifiers. Arrow denotes the index person. Tumor manifestations and age at diagnosis (years) are given below the patient symbol. Carrier status for the germline mutation (of *MSH6* or *MSH2*, see parts (**B**,**C**) below) specific for each family is shown (+, heterozygous carrier; −, non-carrier). F88 additionally had a branch with a germline deletion of *MLH1* exon 16 (plus sign within a circle indicates carriers of this deletion). (**B**) Locations of the *MSH6* germline mutations relative to the functional domains of the MSH6 protein in the MSH2-deficient families F70, F88, and MSH2_9/F286. Exonic structure of *MSH6* is also shown. (**C**) Location of the *MSH2* germline mutation of the MSH6-deficient case MSH6_5 against the gene and protein structure of *MSH2*.

By MS-MLPA, no constitutional epimutations (of *MSH2* or *MSH6* which were the most relevant genes in this context) were detected. To address the hypothesis of *MSH6*-associated predisposition in the nine MSH2-deficient cases that remained without a molecular explanation, *MSH6* was screened by CCP sequencing. A pathogenic germline mutation was discovered in one case: MSH2_9, the index individual from F286, showed a heterozygous *MSH6* c.3013C>T, p.(Arg1005Ter) nonsense mutation (reference sequence NM_000179.2) (Figure 3A,B; Table S2). No obvious second hit (somatic mutation or loss of heterozygosity (LOH)) in *MSH6* was detectable in the patient’s tumor tissue (Table 1). Interestingly, somatic mutation analysis by VarScan 2 revealed a nonsense mutation in *MSH2*, c.2528delG, p.(Cys843fs), with variant allele frequency (VAF) of 11% (Table 1; Table S3). This somatic mutation of *MSH2*, combined with presumed inactivation of the wild-type allele of *MSH2* by a mechanism that remained unknown, likely contributed to the absent MSH2 by IHC.

Case MSH2_1 showed no germline mutation of *MSH6* or other MMR genes. The availability of a tumor sample (CRC) from this patient made it possible to test the hypothesis that MSH2-deficiency resulted from two somatic mutations (“Lynch-like syndrome” [14]). VarScan2 analysis identified a somatic *MSH2* mutation, c.1627G>A, p.(Asp543Asn) (VAF 12%) (Table S3) which was classified as likely pathogenic by Varsome. With relaxed criteria (*p* < 0.05), another somatic *MSH2* mutation, c.2767G>A, p.(Val923Ile), VAF 10%, was present; its pathogenic significance is unknown (VUS by Varsome evaluation). Additionally, a somatic mutation in *MSH6* and two somatic mutations in *MLH1* were detected as well, all missense type and occurring with allele frequencies less than 10%. Sanger sequencing of tumor DNA revealed somatic polymerase proofreading domain mutations (for details, see Section 2.5) which possibly contributed to the multiple somatic MMR gene mutations observed. Collectively, the somatic mutation data justify interpretation of MSH2_1 as a plausible double somatic MMR gene mutation case.

#### 2.3.2. Families from the Hereditary Colorectal Cancer Registry of Finland (LSRFi)

##### F70

Sequencing of leukocyte-derived normal samples from three individuals from F70 (II.5, III.3, III.6) revealed a *MSH6* c.3103C>T, p.(Arg1035Ter) nonsense mutation (Figure 3A,B; Table S2). The mutation affects exon 4, near the start of the lever domain, resulting in the loss of the remaining protein structure. The mutation’s allele frequency is 8.402 × 10^−6^, only found from a non-Finn carrier in the gnomAD database. The mutation has been registered to the InSiGHT database 12 times, being diagnosed in Swedish, Danish, and Dutch patients. The InSiGHT database classifies the variant as class 5. The nonsense mutation was verified with Sanger sequencing and all four tested F70 members were found to be carriers of the mutation (Figure 3A).

Tumors from members of F70 showed variable IHC staining patterns, including MSH6- with positive staining for MSH2 (CRC from II.5), MSH2+, MSH6+ (stomach cancer from III.3), and MSH2-, MSH6- (adenoma from III.6) (Table S1). By CCP screen, the tumor of F70 II.5 revealed a nonsense mutation in *MSH6*: c.2932C>T, p.(Gln978Ter), VAF 22%, serving as a likely “second hit” (Table 1; Table S3). MS-MLPA and MLPA analyses of the normal and tumor samples gave a normal result; there was no observable genomic rearrangement or hypermethylation of *MSH6* in this sample.

By MLPA, the tumor sample of F70 III.6 exhibited a heterozygous deletion in *MSH2-KCNK12-MSH6* region in chromosome 2 presumably involving the wild-type allele (Table 1; Figure S1). The observation was further supported by LOH analysis of deep sequencing data, which displayed LOH of the wild-type allele at the site of the germline mutation. Thus, somatic deletion of the *MSH2-KCNK12-MSH6* region provided a “second hit” to accompany the *MSH6* germline mutation, and additionally, a mechanism for *MSH2* inactivation to explain the IHC pattern (although we could not identify a second somatic “hit”, genetic or epigenetic, in *MSH2*). Tumor samples from F70 showed no instability at the *MSH3*-A_8_ coding region microsatellite sequence.

##### F88

The index of F88 (F88 IV.4) was found to have a 2261 bp deletion (c.1052+2023_c.3557-179, pAsp1058Alafs) covering exons 5 and 6 and part of the flanking introns of *MSH6* (Figure 3A,B; Table S2). Subsequent MLPA analysis was used to verify the deletion. According to the InSiGHT MMR gene variant classification criteria (2016), large deletions in MMR genes are considered class 5, pathogenic.

Incidentally, the sister of the index case (F88 IV.6) was found to be a carrier of *MLH1* exon 16 deletion, based on a genetic test performed in a diagnostic laboratory (Figure 3A). We subsequently investigated the index case (F88 IV.4) and the mother of the sisters (F88 III.5) for *MLH1* exon 16 deletion by MLPA, and the result was negative in the index case and positive in the mother. The mother was also tested for the 2261 bp deletion of *MSH6* and the result was negative. Thus, F88 had two branches with a different genetic deletion. The *MLH1* exon 16 deletion originated from the index case’s mother who transmitted it to the index case’s sister. The *MSH6* exon 5–6 deletion present in the index case may represent de novo alteration, but it is also possible that her father (F88 III.4) had the same deletion; the father’s carrier status could not be determined because no sample was available for testing (Figure 3A).

Endometrial cancer from F88 IV.4 showed MSH2- and MSH6- by IHC. For the second hit analysis, we attempted calculating LOH at the mutation site in the index case’s tumor sample by MLPA, but it was impossible due to poor tumor sample quality. CCP deep sequencing outputs detected no change in the coverage of *MSH6* exons in the tumor. However, a somatic frameshift mutation affecting the coding *MSH6-C_8_* microsatellite sequence was detected: c.3261dupC, p.(Phe1088Leufs) with VAF of 29% (Table 1; Table S3). The observation was verified by Sanger sequencing (Figure S2A). The InSiGHT database classifies *MSH6* p.(Phe1088Leufs) as class 5, pathogenic.

Inspired by the finding of a c.3261dupC, p.Phe1088Leufs frameshift mutation in the coding *MSH6-C_8_* microsatellite in F88 IV.4, coding microsatellite sequences in *MSH2* and *MSH3* were analyzed by CCP and Sanger sequencing, respectively, in an attempt to seek explanations for the observed IHC pattern (MSH2-deficiency). No mutations were observed in the *MSH2-A_7_* microsatellite in F88 (or any other cases with MSH2-deficient tumors in our study series). Sanger sequencing of *MSH3-A_8_* in the tumor from F88 IV.4 showed an indication of deletion of adenine, predicted to result in a frameshift mutation c.1148delA, p.(Lys383Argfs) (Table 1; Figure S2B). No *MSH2* or *MSH6* promoter hypermethylation was observed in the tumor from F88 IV.4.

### 2.4. MSH6 Cohort

MS-MLPA revealed no constitutional epimutations of *MSH6* or *MSH2* among the seven patients with MSH6-deficient tumors. When screened for *MSH2* germline mutations by panel sequencing (CCP), one case (MSH6_5) was found to have a truncating mutation in *MSH2* exon 1: c.110delT, p.(Phe37Serfs*27) (Figure 3A,C; Table S2). Hence, the frequency of *MSH2* germline mutations in patients with MSH6-deficient tumors was 1/7 (14%). Unfortunately, no tumor tissue was available from MSH6_5 for studies of *MSH2* second hit status and possible somatic alterations of *MSH6* to explain the IHC pattern.

### 2.5. Polymerase δ and Ɛ Mutation Analysis

Reduced MMR protein expression can be observed in patients with *POLD1*/*E* proofreading mutations [13]. As CCP does not include the *POLD1* and *POLE* genes, we screened the mutational hotspot exons (*POLD1* exon 11 and *POLE* exons 9 and 13) by Sanger sequencing of the constitutional and tumor tissues from the index individuals from our MSH2- and MSH6-deficient cohorts. No pathogenic or likely pathogenic mutations (according to VarSome predictions [17]) were found except for one case: a somatic nonsense variant in *POLD1* exon 11, c.1453C>T, p.(Gln485Ter) (reference sequence NM_001256849.1) was detected in case MSH2_1 (Figure S3A). This variant is predicted pathogenic by the VarSome database. Additionally, MSH2_1 revealed two variants of unknown significance (VUS) in *POLE* exon 13: c.1324G>A, p.(Glu442Lys), and c.1332G>A, p.(Met444Ile) (reference sequence NM_006231.4) (Figure S3B).

## 3. Discussion

We investigated 60 LS-suspected cases whose cancer susceptibility was unexplained from previous studies. Among 57 index cases examined, a molecular explanation was found for six. The three possible mechanistic scenarios proposed (Section 2.1.; Figure 1) were variably involved. Constitutional epimutation explained one case (MLH1-14) from the MLH1-deficient group (1/38, 3%). Germline mutation in an MMR gene not predicted by the IHC pattern accounted for three cases (MSH2_9/F286 II.2, F70 III.6, and F88 IV.4) from the MSH2-deficient group (3/11, 27%) and one case (MSH6_5) from the MSH6-deficient group (1/7, 14%). Double somatic *MSH2* mutations provided a likely explanation for one MSH2-deficient case (MSH2_1). Finally, a revised classification (from class 3 to class 5) of a previously detected *MSH2* variant established the basis of cancer predisposition for one MSH2-deficient case (MSH2_3).

In pre-screening for LS, patients with absent MSH2 and/or MSH6 protein in tumor tissue constitute the most important group with germline mutations expected, since most consecutive cases lacking MLH1 and PMS2 proteins are due to acquired *MLH1* promoter methylation and isolated PMS2 loss is rare [10,14]. Whenever IHC staining is negative for MSH2 and MSH6, it is customary in the diagnostic setting to examine primarily the *MSH2* gene. This is because *MSH2* mutations outnumber *MSH6* mutations, and the absence of MSH2 subsequently causes the degradation of cellular MSH6 because MSH6 has no other dimerization partners apart from MSH2. A similar relationship exists between MLH1 (primary protein) and PMS2 (secondary protein). Reliance on the instability of the secondary protein when the primary protein is absent has led some to adopt a two-stain method to reduce IHC costs: tumors are first stained with only MSH6 and PMS2, followed by MSH2 and MLH1 stains only if MSH6 or PMS2, respectively, is absent. However, it was found that retained MSH6 expression in the case of *MSH2* germline or double somatic mutations is not uncommon [18], suggesting that the regular four-stain method is preferable. Our investigation was based on the four-stain approach, but this method, too, can be associated with non-canonical patterns, among which the absence of the “wrong” MMR protein is especially challenging for diagnostic purposes. In our investigation 3/11 index individuals with unexplained MSH2-deficient tumors (27%) revealed pathogenic germline variants in *MSH6*, whereas 1/7 individuals with isolated absence of MSH6 (14%) showed a predisposing mutation in *MSH2*. Similarly, in a study by Mensenkamp et al. [14], 2 of 17 patients (12%) with MSH2-deficient tumors not explained by *MSH2* or *EPCAM* mutations in the germline, had a predisposing mutation in *MSH6*, and 1 of 5 unexplained cases with isolated MSH6 loss (20%) was attributable to a germline mutation in *MSH2.* In another investigation [10], universal screening on 500 consecutive CRCs resulted in the diagnosis of LS in 18 individuals, among which IHC findings were discordant with the germline mutation results in two, including one with germline mutation in *MSH6* although all four MMR proteins were expressed, and one with a germline mutation in *MSH6* (p.P1309SfsX11) although the simultaneous lack of MSH2 and MSH6 proteins pointed to an *MSH2* mutation.

Our study suggests that heterozygous germline *MSH6* mutations may result in shortage and aberrant function of MSH2 protein in several possible ways (Table 1) including: somatic deletion of the *MSH2-MSH6* region (as seen in F70 III.6), combined loss of *MSH6* and *MSH3* in somatic tissue (as seen in F88 IV.4), or hindered MSH2 dimerization caused by the loss of the COOH-terminal interaction domain in the truncated MSH6 proteins (applies to the germline *MSH6* mutations of F70, F88, and F286, and the *MSH6* mutation reported by Hampel et al. [10]). Alternatively, the truncated MSH6 protein may be able to bind to MSH2 and/or the DNA but lack any repair activity, thus competing with wild-type MSH2 and MSH6. Our observations from F88 are analogous to findings reported by Morak et al. [19]. They described a case where a joint absence of MSH2 and MSH6 likely resulted from the simultaneous lack of MSH3 and MSH6 proteins, the binding partners of MSH2, due to germline and somatic inactivating events. Their case exhibited a truncating heterozygous *MSH6* variant and a truncating heterozygous *MSH3* variant in the germline, accompanied by LOH at the *MSH6* region and somatic heterozygous frameshift changes affecting *MSH6-C_8_* and *MSH3-A_8_* coding repeats. Our case (F88 IV.4) showed a heterozygous germline mutation of *MSH6* (genomic deletion of exons 5 and 6), combined with somatic frameshift mutations *MSH6-C_8_* and *MSH3-A_8_* (Table 1).

Interestingly, despite the positive MSH2 staining of the tumor nuclei in MSH6_5, a germline mutation of *MSH2* p. (Phe37Serfs*27) was observed. MSH2 positivity could imply the absence of a somatic “second hit”, which would also be compatible with stable microsatellites in the patient’s endometrial cancer (Table S1). Experience from colorectal adenomas from patients with LS shows that tumor development is possible when the wild-type allele is still present, and the respective protein is expressed [20]. However, almost all CRCs and most endometrial carcinomas from LS patients lack the protein corresponding to the gene mutant in the germline [6]. Furthermore, MSH6 negativity of the tumor from MSH6_5 would technically indicate that a second hit in *MSH2* would be expected. Theoretically, two somatic mutations in *MSH6* are possible. Unavailability of tumor tissue from this patient unfortunately prevented a closer molecular investigation into this issue.

Constitutional epimutations of *MLH1* (primary or secondary) and *MSH2* (secondary to *EPCAM* 3′-end deletions) may account for a few percent of Lynch-suspected cases without conventional genetic mutations despite abnormal IHC [21,22]. We found one case (MLH1_14) with an apparently primary constitutional epimutation of *MLH1* among 38 MLH1-, PMS2-deficient cases tested (3%). We previously screened for constitutional epimutations a LS-suspected series ascertained in an analogous manner and detected two positive cases (one primary and one secondary epimutation of *MLH1*) among 22 cases examined (9%) [21]. Thus, the combined proportion of constitutional *MLH1* epimutations in LS-suspected cases with MLH1-deficient tumors is 3/60 (5%). As case MLH1_14 illustrates, carriers of constitutional epimutations clinically resemble carriers of conventional genetic mutations in MMR genes. However, family features and transmission pattern make a distinction [23]. Primary constitutional epimutations are seldom associated with any marked family history of cancer (in agreement, MLH1_14 had no close relatives diagnosed with cancers of the LS spectrum) and are usually not transmitted from parent to child. In contrast, secondary epimutations of *MLH1* or *MSH2* give rise to classical LS families and follow dominant Mendelian inheritance. *MSH2* was not affected by constitutional epimutations in our MSH2-deficient cases (*EPCAM* deletions were excluded by MLPA from the outset). No constitutional epimutations of *MSH6* or *PMS2* were detected, which complies with findings from another population [24]. Moreover, we found no evidence of hypermethylation of *MSH2* or *MSH6* promoters as possible second hits in our MSH2- or MSH6-deficient tumors.

Occasionally, defective MMR protein expression in tumor tissue can be secondary to germline or somatic mutation in another DNA repair or replication-associated gene, such as *POLD1/E* [13] or less frequently, *MUTYH* [25]. In a study by Jansen et al. [13], 9 of 62 suspected LS patients (15%) with abnormal IHC and/or MSI, but no identifiable MMR gene germline mutations, had germline (2/9) or somatic (7/9) variants in the *POLD1/E* exonuclease domain. With one exception (case MSH6_7 with insufficient DNA), we Sanger sequenced the mutational hotspots exons in *POLD1/E* (see Section 4) in normal tissues from all our cases suspected of having *MSH2* or *MSH6* mutation, and in available tumor tissues as well when relevant (a separate analysis was necessary since the CCP panel did not include the *POLD1/E* genes). No germline *POLD1/E* mutations were found, but one pathogenic variant and two VUS were observed in the tumor of MSH2_1. Morak et al. [25] screened *MUTYH* in 85 patients with abnormal IHC but no detectable germline mutations in MMR genes: one patient (1%) showed a biallelic *MUTYH* germline mutation, accompanied by two somatic *MSH2* mutations in a MSH2/MSH6-deficient tumor (a sebaceous gland carcinoma). All our *MSH2-* and *MSH6-*suspect cases were screened for germline *MUTYH* mutations as part of the CCP or whole-genome sequencing and no positive cases were found. No obviously pathogenic germline mutations beyond the predisposing MMR genes were identified in other DNA repair genes included in CCP (Table S2).

Double somatic mutations of MMR genes may account for over half of MMR-deficient tumors with neither somatic *MLH1* promoter methylation nor germline mutations in MMR genes [14,26]. Results from our population-based investigation conducted earlier [26] are compatible with this notion, but we could not formally test this possibility in the present series since the tumor tissues available to us originated from germline mutation carriers. MSH2_1 with no germline mutation found constituted the single exception. This case could be attributed to two somatic *MSH2* mutations, provided that the mutations affected different alleles (which cannot be resolved by our current assays).

Cohorts of consecutive LS-suspected cases which referred to clinical and basic genetic evaluation over a decade constitute a strength of this investigation. Our comprehensive approach (genetic and epigenetic) to identify the mechanisms of cancer predisposition in patients is another advantage. The relatively modest numbers of cases in certain groups (e.g., the MSH6-deficient group) and poor availability (and sometimes low quality due to formalin-fixed paraffin-embedded (FFPE)-origin) of tumor samples are among the limitations. Lack of tumor tissues made a comprehensive evaluation of MLH1-deficient cases impossible. In analogy to our observations from MSH2- and MSH6-deficient cases, existing literature [14] suggests that small proportions of MLH1-deficient cases as well as cases with solitary PMS2-deficiency exhibit germline mutations in MMR genes not predicted by the IHC pattern. Another limitation caused by unavailability of tumor tissues was that we could not determine the frequency of cases with double somatic mutations of MMR genes in our cohorts, as pointed out above. Finally, the mechanisms we tested as possible explanations for altered MMR protein expression are by no means all-inclusive. For example, microRNAs (e.g., overexpression of miR-155 can downregulate MLH1 and MSH2 [27]), and post-translational modifications (phosphorylation of MSH2 increases resistance to ubiquitin-induced protein degradation [28]) have been identified as somatic regulators of MMR gene expression.

## 4. Materials and Methods

### 4.1. Patients and Samples

Our patient material consisted of research and clinic-based cohorts (Figure 1; Table S1). The patients represented putative LS families from diagnostic units across Finland (*n* = 55). In addition, five individuals originated from two large families (F70 and F88) from the Hereditary Colorectal Cancer Registry of Finland (LSRFi). The institutional review board of the Helsinki University Hospital approved this study (466/E6/01). All the samples and patient information were obtained and used according to the guidelines of this approval and following a written consent from the patients. The National Supervisory Authority for Welfare and Health (Dnro 1272/04/044/07 and Dnro 10741/06.01.03.01/2015) approved the collection of archival specimens.

The conventional diagnostic screening protocol consisted of IHC staining of tumor tissues for MLH1, PMS2, MSH2, and MSH6 proteins, occasionally supplemented with MSI analysis, which were followed by Sanger sequencing of the coding regions and immediate exon/intron borders of the gene primarily suspected to be affected in each case based on the IHC pattern [7]. An accredited diagnostic laboratory at Helsinki University Hospital (HUSLAB), highly experienced in such analyses, performed the IHC, MSI, and sequencing assays. If no pathogenic or likely pathogenic germline alteration was found, MLPA was performed in our research laboratory. Cases/families remaining mutation-negative after all these assays were eligible for this study. Fresh frozen and/or formalin-fixed paraffin-embedded (FFPE) specimens of tumor and matching normal tissues were collected from pathology departments of different hospitals and used for IHC analysis and DNA extraction. Areas with pure normal or high tumor percentages, with minimal intervening stroma or inflammatory cells, were selected and verified histologically and subsequently dissected out for DNA preparation. DNA was extracted from EDTA blood or FFPE–derived tissues of the patients according to the protocols used by Renkonen et al. [29] and Isola et al. [30].

### 4.2. Immunohistochemical Staining (IHC)

IHC staining analyses were conducted on FFPE samples by HUSLAB Laboratory of Pathology as described by Renkonen et al. [29]. The analyses included positive controls and each slide was required to have sufficient representation of normal and malignant tissue as tests for analysis coherence. Antibodies used were anti-MLH1 (clone G168-15; Pharmingen, San Diego, CA, USA), anti-MSH2 (clone FE-11; Calmiochem/Oncogene Research, San Diego, CA, USA), anti-MSH6 (clone 44; Transduction Laboratories, San Diego, CA, USA), and anti-PMS2 (clone 556415; BD Pharmingen, Franklin Lakes, NJ, USA). Both MLH1 and MSH6 antibodies were full-length, monoclonal antibodies, whereas MSH2 antibody was monoclonal, raised against carboxyl-terminal fragment of the human MSH2 protein. For visualization of the samples, EnVision+ System (DakoCytomation, Glostrump, Denmark) was applied. A pathologist evaluated all samples in this study for nuclear MMR protein staining. If any of the tumor cells displayed positive nuclear staining, the sample was considered positive for the tested protein (as instructed by the International Collaborative Group on HNPCC).

### 4.3. Microsatellite Instability Analysis (MSI)

The tumors’ MSI status was determined using fluorescent markers BAT25 and BAT26, and sometimes additionally D5S349, D2S123, and D17S250. MSI-high status required that at least one of the mononucleotide repeat markers, BAT25 and BAT26, showed MSI [31].

### 4.4. MMR Gene Methylation Status

To determine the methylation status changes in the MMR genes of both normal and tumor samples, MS-MLPA was performed by using the SALSA MLPA ME011-B3 probemix (MRC-Holland, Amsterdam, Netherlands) according to the manufacturer’s instructions and as described in Gylling et al. [21].

### 4.5. MMR Gene Copy Number Status

To evaluate the possibility of copy number changes in the MMR genes, MLPA was applied. SALSA MLPA P003-D1 and SALSA MLPA P072-C1 (MRC-Holland, Amsterdam, Netherlands) were used for *MLH1*/*MSH2* and *MSH6*, respectively. The probemixes used in (MS) MLPA include probes testing for sample fragmentation and sufficient digestion.

Fragment analysis for both MS-MLPA and MLPA was carried out using on ABI3730xl DNA Analyzer (Applied Biosystems, Foster City, CA, USA) at the Institute for Molecular Medicine of Finland (FIMM) Technology Centre and analyzed on GeneMapper software version 5.0 (Thermo Fisher, Waltham, MA, USA) and Coffalyser^TM^ (MRC-Holland, Amsterdam, Netherlands).

### 4.6. Sanger Sequencing

To determine the possible loss of MMR gene expression in tumor tissue due to mutations in genes encoding for polymerase Ɛ and δ, mutation hotspots in *POLE* exons 9 and 13, as well as *POLD1* exon 11 were Sanger sequenced in constitutional and tumor tissues using primers by Church et al. [32] and Valle et al. [33], respectively.

Custom primers for microsatellite repeat sequences *MSH6-C_8_* and *MSH3-A_8_* were designed using Primer3 software (bioinfo.ut.ee). Primers were as follows: *MSH6-C_8_*-F: 5′ GGGTGATGGTCCTATGTGTC 3′, *MSH6-C_8_*-R: 3′ CGTAATGCAAGGATGGCGT 5′, and *MSH3-A_8_*-F: 5′ ATGTGAATCCCCTAATCAAGCTGG 3′, *MSH3-A_8_*-R: 3′ GCAAAGTACTTACCACAATGCCAATAAAA 5′.

### 4.7. Deep Sequencing and Variant Analysis

Whole genome sequencing was applied to leukocyte-derived DNA from three cases from the research families (F70 and F88). The libraries were prepared using the ThruPLEX^®^ DNA-seq kit (Rubicon Genomics, Ann Arbor, MI, USA) and run on Illumina HiSeq 2500 platform (San Diego, CA, USA).

Sixteen normal samples and four matching tumor samples were analyzed using CCP. ThruPLEX^®^ DNA-seq kit (Rubicom Genomics), KAPA HyperPlus kit (Roche, Basel, Switzerland), or Accel-NGS 1S Plus (Swift Biosciences, Ann Arbor, MI, USA) was used for library preparation. Roche/NimbleGen’s SeqCap EX Exome Library v2.0 is an exome enrichment platform using biotinylated oligonucleotide baits which are complementary to the exome targets. These baits hybridize to sequencing libraries prepared from fragmented genomic DNA. CCP analysis uses 2.1 million overlapping probes to cover 578 cancer-associated genes. One tumor sample was processed with Twist Core Exome Kit and the library were prepared using Twist EF library preparation protocol and run on NovaSeq S1 PE101.

FIMM Next Generation Sequencing service performed the primary (read preparation and quality control) and secondary analysis (copy number variant (CNV) calls) on the samples to produce ready-to-analyze whole genome sequencing (WGS) data. The sequencing data was analyzed by in-house variant calling pipeline (VCP) versions v3.3 and v3.4. (WGS), v3.7b (exome sequencing (WES)), 3.6 and 3.7 (CCP). The VCP analysis includes quality control of raw reads pre- and post-alignment, pre-processing for sequencing artifacts, alignment to human reference genome (GRCh37) with the Burrows–Wheeler alignment (BWA) [34], and variant calling with SAMtools package [35]. Performance characteristics of all deep sequencing experiments included in this study are given in Table S4.

Tertiary analysis was carried out with VarSeq^®^ (v1.3.2, Golden Helix) and analyzed according to the principles used by Olkinuora et al. [5]. Briefly, only high-quality (Phred-scale likelihood >70), rare (variant allele frequency <0.003 in the ExAC and GnomAD databases), and nonsynonymous variants (frameshift, stop gained/lost, missense, disrupting donor/acceptor site variants) were considered. Additionally, only variants predicted tolerated by a maximum of 1 in silico software (SIFT, PolyPhen2 HVAR, MutationTaster, MutationAssessor, FATHMM, and FATHMM MKL) were taken for further analysis. Only variants affecting genes participating in DNA repair according to Wood et al. [36] were considered.

### 4.8. Somatic Mutation Profiling

VarScan2 mutation detection algorithm version 2.3.2 was carried out by FIMM for identifying somatic, non-synonymous mutations from the tumor-normal sample pairs. The following mutation calling parameters were used: normal-purity 1, strand-filter 1, min coverage 8 and 6 (for normal and tumor samples, respectively), minimum variant frequency 0.005, and somatic *p*-value 1. Annotation of the mutations was done using SnpEff version 4.0 with the Ensembl v68 annotation database. Variants with a somatic *p*-value less than 0.01 were selected for further analyses.

### 4.9. Loss of Heterozygosity (LOH) Analysis

LOH analysis for relevant MMR genes was based on the comparison of deep sequencing data for paired tumor and normal samples [37] and was performed by VarSeq (GoldenHelix^®^, Bozeman, MT, USA). We followed the thresholds set for putative and strict LOH in Ollikainen et al. [38]. When reporting LOH data, putative and strict LOH are called LOH for simplicity.

## 5. Conclusions

Two main conclusions directly relevant for LS diagnostics can be drawn from our findings. First, as the predisposing mutation was in *MSH6* in 27% of index cases with MSH2-deficient tumors, and in *MSH2* in 14% of cases with solitary absence of MSH6, germline mutation analysis in such cases should include both *MSH2* and *MSH6* and not just the gene primarily suspected from the IHC pattern. Multigene panel sequencing, which is gradually replacing single-gene tests in LS diagnostics, is expected to alleviate this problem. Panel testing would not be affected by misleading IHC patterns since all main MMR genes (and other relevant cancer predisposition genes, too, as appropriate) can be interrogated simultaneously. Second, in MLH1-deficient cases with no point mutations or large rearrangements of *MLH1*, the possibility of constitutional epimutations should be kept in mind (5% frequency in the combined series of this and our previous investigation [21]). Although constitutional epimutations are rare, awareness of their existence and unique characteristics, including variable patterns of transmission, is important for a proper clinical management of the patients and their relatives.

## Figures and Tables

**Table 1:** Summary of germline and somatic alterations in patients from the MSH2-deficient cohort with tumor samples available for molecular studies.

Case ID	IHC Pattern	Germline Mutation	Second Hit	Additional Somatic Alterations Relevant for IHC Pattern
MSH2_9/F286 II.2	MSH2-, MSH6-	*MSH6* c.3013C>T,	None identified	*MSH2* c.2528delG,
		p.(Arg1005Ter) *		p.(Cys843fs)
				
F70 III.6 (index)	MSH2-, MSH6-	*MSH6* c.3103C>T,	*MSH2*-*KCNK12*-*MSH6* deletion	*(MSH2*-*KCNK12*-*MSH6* deletion)
		p.(Arg1035Ter)*		
				
F70 II.5	MSH6-	*MSH6* c.3103C>T,	*MSH6* c.2932C>T,	Not applicable
		p.(Arg1035Ter)*	p.(Gln978Ter)	
				
F88 IV.4	MSH2-, MSH6-	*MSH6* c.1052+2023_c.3557-179,	*MSH6* c.3261dupC,	*MSH3* c.1148delA,
		p.(Asp1058Alafs)	p.(Phe1088Leufs) *	(pLys383Argfs)

An asterisk (*) denotes the variants present in the InSiGHT database.

## Supplementary Materials

The following are available online at https://www.mdpi.com/2072-6694/12/7/1853/s1, Figure S1. Schematic of the *MSH2-KCNK12-MSH6* deletion in F70 III.6; Figure S2. Chromatograms of the coding microsatellite repeats in F88 IV.4; Figure S3. Somatic polymerase proofreading domain mutations in MSH2_1; Table S1. Clinical and IHC/MSI data, and assays performed; Table S2. Germline mutations in DNA repair genes in all cases sequenced; Table S3. Significant somatic mutations by VarScan2 analysis; Table S4. Performance characteristics of the deep sequencing runs in this study.

## References

[1] Arnold M., Sierra M.S., Laversanne M., Soerjomataram I., Jemal A., Bray F. (2017). Global Patterns and Trends in Colorectal Cancer Incidence and Mortality. Gut.

[2] Lynch H.T., Snyder C.L., Shaw T.G., Heinen C.D., Hitchins M.P. (2015). Milestones of Lynch Syndrome: 1895–2015. Nat. Rev. Cancer.

[3] Thompson B.A., Spurdle A.B., Plazzer J., Greenblatt M.S., Akagi K., Al-Mulla F., Bapat B., Bernstein I., Capellá G., Den Dunnen J.T. (2013). Application of a Five-Tiered Scheme for Standardized Classification of 2,360 Unique Mismatch Repair Gene Variants Lodged on the InSiGHT Locus-Specific Database. Nat. Genet..

[4] Adam R., Spier I., Zhao B., Kloth M., Marquez J., Hinrichsen I., Kirfel J., Tafazzoli A., Horpaopan S., Uhlhaas S. (2016). Exome Sequencing Identifies Biallelic MSH3 Germline Mutations as a Recessive Subtype of Colorectal Adenomatous Polyposis. Am. J. Hum. Genet..

[5] Olkinuora A., Nieminen T.T., Mårtensson E., Rohlin A., Ristimäki A., Koskenvuo L., Lepistö A., Gebre-Medhin S., Nordling M., Peltomäki P. (2018). Biallelic Germline Nonsense Variant of MLH3 Underlies Polyposis Predisposition. Genet. Med..

[6] Peltomäki P. (2016). Update on Lynch Syndrome Genomics. Fam. Cancer.

[7] Hendriks Y.M.C., de Jong A.E., Morreau H., Tops C.M.J., Vasen H.F., Wijnen J.T., Breuning M.H., Brocker-Vriends A.H.J.T. (2006). Diagnostic Approach and Management of Lynch Syndrome (Hereditary Nonpolyposis Colorectal Carcinoma): A Guide for Clinicians. CA Cancer J. Clin..

[8] Rubenstein J.H., Enns R., Heidelbaugh J., Barkun A. (2015). American Gastroenterological Association Institute Guideline on the Diagnosis and Management of Lynch Syndrome. Gastroenterology.

[9] Stjepanovic N., Moreira L., Carneiro F., Balaguer F., Cervantes A., Balmaña J., Martinelli E. (2019). Hereditary Gastrointestinal Cancers: ESMO Clinical Practice Guidelines for Diagnosis, Treatment and Follow-Up. Ann. Oncol..

[10] Hampel H., Frankel W.L., Martin E., Arnold M., Khanduja K., Kuebler P., Clendenning M., Sotamaa K., Prior T., Westman J.A. (2008). Feasibility of Screening for Lynch Syndrome among Patients with Colorectal Cancer. J. Clin. Oncol..

[11] Moreira L., Balaguer F., Lindor N., de la Chapelle A., Hampel H., Aaltonen L.A., Hopper J.L., Le Marchand L., Gallinger S., Newcomb P.A. (2012). Identification of Lynch Syndrome Among Patients With Colorectal Cancer. JAMA.

[12] Loughrey M., Waring P., Tan A., Trivett M., Kovalenko S., Beshay V., Young M.A., McArthur G., Boussioutas A., Dobrovic A. (2007). Incorporation of Somatic BRAF Mutation Testing into an Algorithm for the Investigation of Hereditary Non-Polyposis Colorectal Cancer. Fam. Cancer.

[13] Jansen A.M., Wezel T., Van Den Akker B.E., Ventayol Garcia M., Ruano D., Tops C., Wagner A., Letteboer T., Gómez García E., Devilee P. (2016). Combined Mismatch Repair and POLE/POLD1 Defects Explain Unresolved Suspected Lynch Syndrome Cancers. Eur. J. Hum. Genet..

[14] Mensenkamp A.R., Vogelaar I.P., van Zelst–Stams W.A.G., Goossens M., Ouchene H., Hendriks–Cornelissen S.J.B., Kwint M.P., Hoogerbrugge N., Nagtegaal I.D., Ligtenberg M.J.L. (2014). Somatic Mutations in MLH1 and MSH2 are a Frequent Cause of Mismatch-Repair Deficiency in Lynch Syndrome-Like Tumors. Gastroenterology.

[15] Deng G., Chen A., Hong J., Chae H.S., Kim Y.S. (1999). Methylation of CpG in a Small Region of the hMLH1 Promoter Invariably Correlates with the Absence of Gene Expression. Cancer Res..

[16] Deng G., Chen A., Pong E., Kim Y.S. (2001). Methylation in hMLH1 Promoter Interferes With Its Binding to Transcription Factor CBF and Inhibits Gene Expression. Oncogene.

[17] Kopanos C., Tsiolkas V., Kouris A., Chapple C.E., Aguilera M.A., Meyer R., Massouras A. (2019). VarSome: The Human Genomic Variant Search Engine. Bioinformatics.

[18] Pearlman R., Markow M., Knight D., Chen W., Arnold C.A., Pritchard C.C., Hampel H., Frankel W.L. (2018). Two-Stain Immunohistochemical Screening for Lynch Syndrome in Colorectal Cancer may Fail to Detect Mismatch Repair Deficiency. Mod. Pathol..

[19] Morak M., Käsbauer S., Kerscher M., Laner A., Nissen A., Benet-Pagès A., Schackert H., Keller G., Massdorf T., Holinski-Feder E. (2017). Loss of MSH2 and MSH6 due to Heterozygous Germline Defects in MSH3 and MSH6. Fam. Cancer.

[20] Mäki-Nevala S., Valo S., Ristimäki A., Sarhadi V., Knuutila S., Nyström M., Renkonen-Sinisalo L., Lepistö A., Mecklin J., Peltomäki P. (2019). DNA Methylation Changes and Somatic Mutations as Tumorigenic Events in Lynch Syndrome-Associated Adenomas Retaining Mismatch Repair Protein Expression. EBioMedicine.

[21] Gylling A., Ridanpää M., Vierimaa O., Aittomäki K., Avela K., Kääriäinen H., Laivuori H., Pöyhönen M., Sallinen S., Wallgren-Pettersson C. (2009). Large Genomic Rearrangements and Germline Epimutations in Lynch Syndrome. Int. J. Cancer.

[22] Niessen R.C., Hofstra R.M., Westers H., Ligtenberg M.J.L., Kooi K., Jager P.O., de Groote M.L., Dijkhuizen T., Olderode-Berends M.J., Hollema H. (2009). Germline Hypermethylation of MLH1 and EPCAM Deletions are a Frequent Cause of Lynch Syndrome. Genes Chromosomes Cancer.

[23] Hitchins M.P. (2015). Constitutional epimutation as mechanism for cancer causality and heritability?. Nat. Rev. Cancer.

[24] Liu Y., Chew M.H., Goh X.W., Tan S.Y., Loi C.T.T., Tan Y.M., Law H.Y., Koh P.K., Tang C.L. (2014). Systematic Study on Genetic and Epimutational Profile of a Cohort of Amsterdam Criteria-Defined Lynch Syndrome in Singapore. PLoS ONE.

[25] Morak M., Heidenreich B., Keller G., Hampel H., Laner A., de la Chapelle A., Holinski-Feder E. (2014). Biallelic MUTYH Mutations can Mimic Lynch Syndrome. Eur. J. Hum. Genet..

[26] Porkka N., Lahtinen L., Ahtiainen M., Böhm J.P., Kuopio T., Eldfors S., Mecklin J., Seppälä T.T., Peltomäki P. (2019). Epidemiological, Clinical and Molecular Characterization of Lynch-like Syndrome: A Population-based Study. Int. J. Cancer.

[27] Valeri N., Gasparini P., Fabbri M., Braconi C., Veronese A., Lovat F., Adair B., Vannini I., Fanini F., Bottoni A. (2010). Modulation of Mismatch Repair and Genomic Stability by miR-155. Proc. Natl. Acad. Sci. USA.

[28] Hernandez-Pigeon H., Quillet-Mary A., Louat T., Schambourg A., Humbert O., Selves J., Salles B., Laurent G., Lautier D. (2005). hMutSα is Protected from Ubiquitin-Proteasome-Dependent Degradation by Atypical Protein Kinase Cζ Phosphorylation. J. Mol. Biol..

[29] Renkonen E., Zhang Y., Lohi H., Salovaara R., Abdel-Rahman W., Nilbert M., Aittomaki K., Jarvinen H., Mecklin J., Lindblom A. (2003). Altered Expression of MLH1, MSH2, and MSH6 in Predisposition to Hereditary Nonpolyposis Colorectal Cancer. J. Clin. Oncol..

[30] Isola J., DeVries S., Chu L., Ghazvini S., Waldman F. (2014). Analysis of Changes in DNA Sequence Copy Number by Comparative Genomic Hybridization in Archival Paraffin-Embedded Tumor Samples. Am. J. Pathol..

[31] Loukola A., Eklin K., Laiho P., Salovaara R., Kristo P., Jarvinen H., Mecklin J., Launonen V., Aaltonen L.A. (2001). Microsatellite Marker Analysis in Screening for Hereditary Nonpolyposis Colorectal Cancer (HNPCC). Cancer Res..

[32] Church D.N., Stelloo E., Nout R.A., Valtcheva N., Depreeuw J., ter Haar N., Noske A., Amant F., Tomlinson I.P.M., Wild P.J. (2015). Prognostic Significance of POLE Proofreading Mutations in Endometrial Cancer. J. Natl. Cancer Inst..

[33] Valle L., Hernández-Illán E., Bellido F., Aiza G., Castillejo A., Castillejo M., Navarro M., Seguí N., Vargas G., Guarinos C. (2014). New Insights into POLE and POLD1 Germline Mutations in Familial Colorectal Cancer and Polyposis. Hum. Mol. Genet..

[34] Li H., Durbin R. (2009). Fast and Accurate Short Read Alignment with Burrows-Wheeler Transform. Bioinformatics.

[35] Li H., Handsaker B., Wysoker A., Fennell T., Ruan J., Homer N., Marth G., Abecasis G., Durbin R. (2009). The Sequence Alignment Map Format and SAMtools. Bioinformatics.

[36] Wood R.D., Mitchell M., Sgouros J., Lindahl T. (2001). Human DNA Repair Genes. Science.

[37] Porkka N., Valo S., Nieminen T.T., Olkinuora A., Mäki-Nevala S., Eldfors S., Peltomäki S. (2017). Sequencing of Lynch Syndrome Tumors Reveals the Importance of Epigenetic Alterations. Oncogene.

[38] Ollikainen M., Abdel-Rahman W.M., Moisio A., Lindroos A., Kariola R., Järvelä I., Pöyhönen M., Butzow R., Peltomäki P. (2005). Molecular Analysis of Familial Endometrial Carcinoma: A Manifestation of Hereditary Nonpolyposis Colorectal Cancer Or a Separate Syndrome?. J. Clin. Oncol..

