# Supplementary figures and images for: Molecular Basis of Mismatch Repair Protein Deficiency in Tumors from Lynch Suspected Cases with Negative Germline Test Results
Source: Cancers (Basel). 2020 Jul 9;12(7):1853. doi: 10.3390/cancers12071853 (PMC7408769; doi:10.3390/cancers12071853)

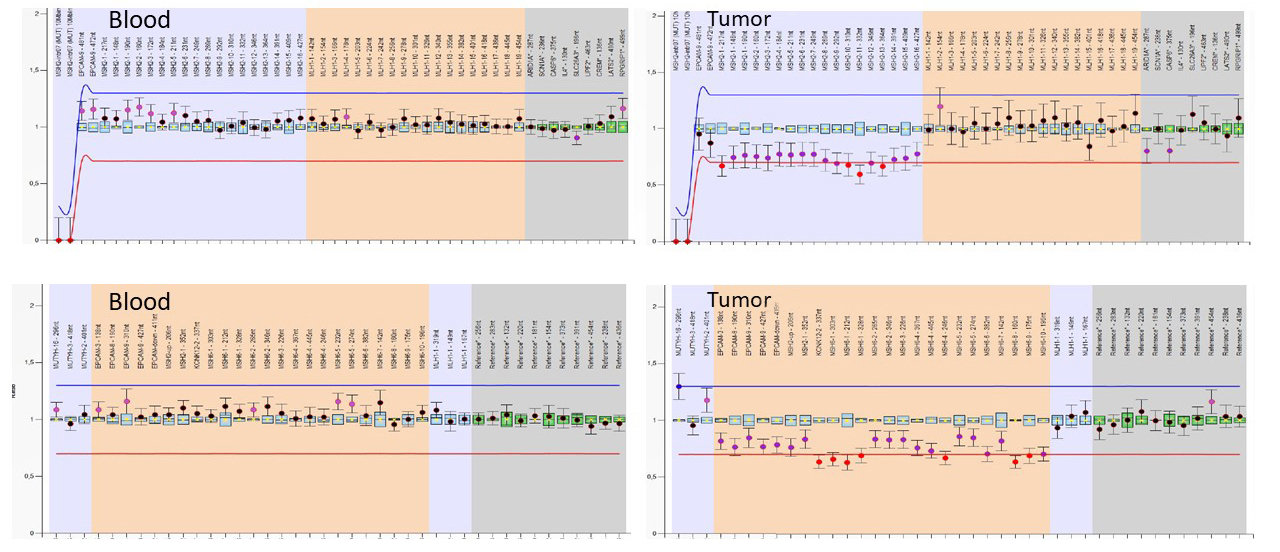

Supplement: Supplementary file 1 [file cancers-12-01853-s001.zip › Supplementary_Files_olkinuora/Figure_S1_olkinuora.jpg]

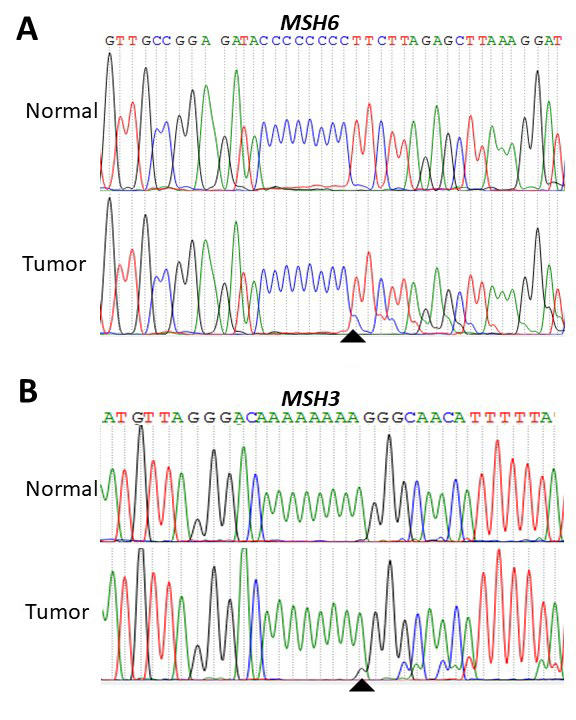

Supplement: Supplementary file 1 [file cancers-12-01853-s001.zip › Supplementary_Files_olkinuora/Figure_S2_olkinuora.jpg]

**A**

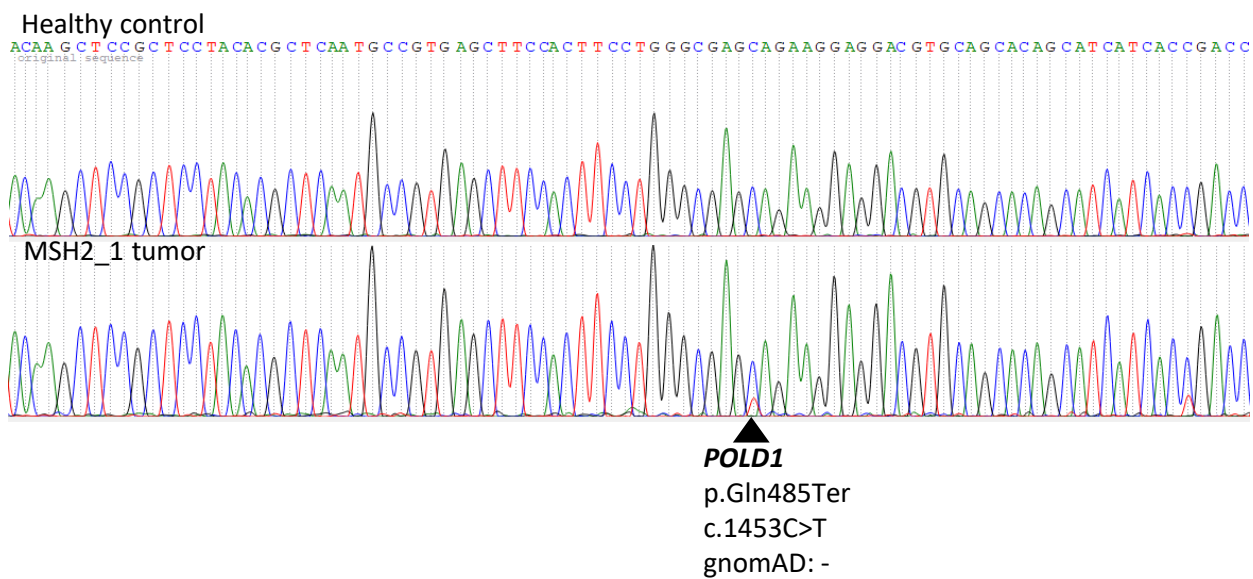

**B**

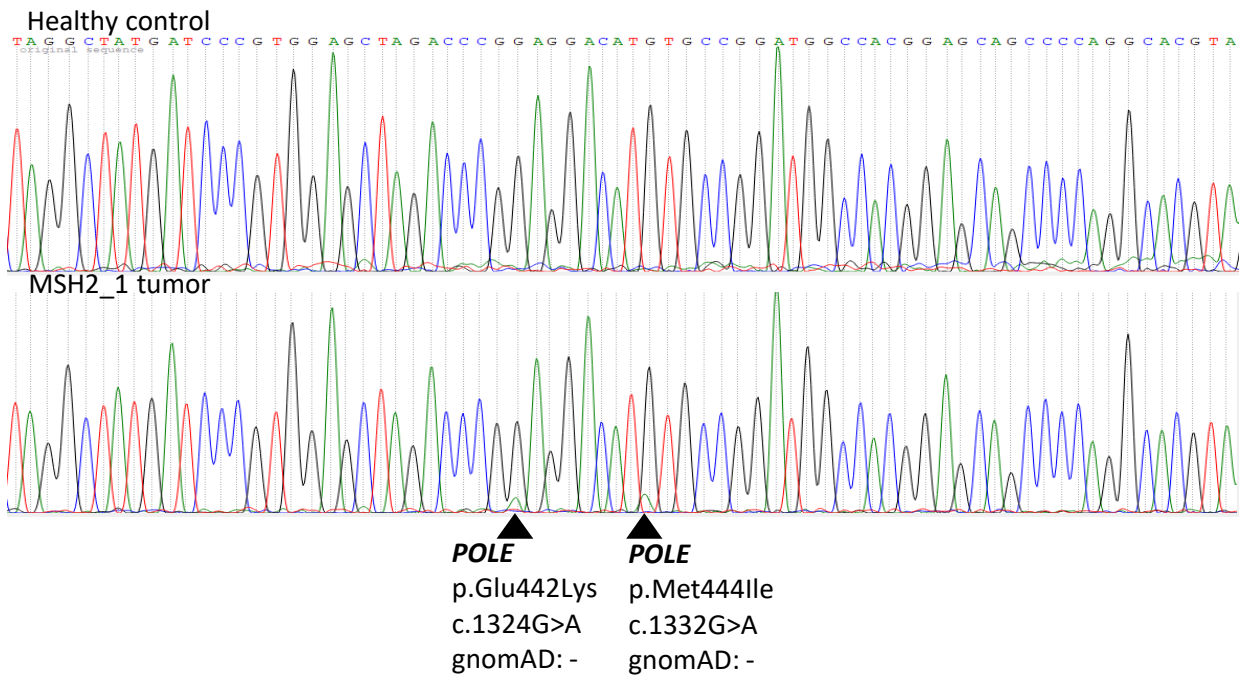

Supplement: Supplementary file 1 [file cancers-12-01853-s001.zip › Supplementary_Files_olkinuora/Figure_S3_olkinuora.pdf]
